# AI co-pilot: content-based image retrieval for the reading of rare diseases in chest CT

**DOI:** 10.1038/s41598-023-29949-3

**Published:** 2023-03-16

**Authors:** Johannes Haubold, Ke Zeng, Sepehr Farhand, Sarah Stalke, Hannah Steinberg, Denise Bos, Mathias Meetschen, Anisa Kureishi, Sebastian Zensen, Tim Goeser, Sandra Maier, Michael Forsting, Felix Nensa

**Affiliations:** 1 Department of Diagnostic and Interventional Radiology and Neuroradiology, University Hospital Essen, Hufelandstraße 55, 45147 Essen, Germany; 2 Siemens Medical Solutions Inc., Malvern, PA USA; 3 Institute of Artificial Intelligence in Medicine, University Hospital Essen, Hufelandstraße 55, 45147 Essen, Germany; 4 Georg Thieme Verlag KG, Stuttgart, Germany; 5 Department of Radiology and Neuroradiology, Kliniken Maria Hilf, Viersener Str. 450, 41063 Mönchengladbach, NRW Germany

**Keywords:** Computed tomography, Medical imaging

## Abstract

The aim of the study was to evaluate the impact of the newly developed Similar patient search (SPS) Web Service, which supports reading complex lung diseases in computed tomography (CT), on the diagnostic accuracy of residents. SPS is an image-based search engine for pre-diagnosed cases along with related clinical reference content (https://eref.thieme.de). The reference database was constructed using 13,658 annotated regions of interest (ROIs) from 621 patients, comprising 69 lung diseases. For validation, 50 CT scans were evaluated by five radiology residents without SPS, and three months later with SPS. The residents could give a maximum of three diagnoses per case. A maximum of 3 points was achieved if the correct diagnosis without any additional diagnoses was provided. The residents achieved an average score of 17.6 ± 5.0 points without SPS. By using SPS, the residents increased their score by 81.8% to 32.0 ± 9.5 points. The improvement of the score per case was highly significant (p = 0.0001). The residents required an average of 205.9 ± 350.6 s per case (21.9% increase) when SPS was used. However, in the second half of the cases, after the residents became more familiar with SPS, this increase dropped to 7%. Residents’ average score in reading complex chest CT scans improved by 81.8% when the AI-driven SPS with integrated clinical reference content was used. The increase in time per case due to the use of the SPS was minimal.

## Introduction

The differentiation between complex lung diseases requires a high level of expertise. Thus, in the case of even the most common pulmonary fibrosis, the idiopathic pulmonary fibrosis (IPF), patient care is greatly hindered by a high number of incorrect diagnoses and a median delay in treatment of one year^1^. Recent studies show that convolutional neural networks have great potential in assisting the detection of different lung patterns in 2D patches of interstitial lung diseases (ILDs), including patterns such as ground-glass opacities, micronodules, consolidation, reticulation, and honeycombing^2–5^. The accuracy of radiologists’ diagnoses is thus likely to improve with the incorporated use of convolution neural networks in the diagnostic process^6^. Nevertheless, knowing individual patterns is often not sufficient for the final diagnosis since individual patterns such as ground glass opacities occur in a variety of diseases.

Alternatively, radiologists can compare the case with digital databases of reference cases to find the correct diagnosis^7^. However, this is likely to be time-consuming. A possible solution to combine the advantages of a reference database with modern AI-based image analysis technologies is a content-based image retrieval system^8^. This is used to compare the similarity of images based on extracted image features. The images with similar patterns in the reference database are subsequently assigned to the image. Content-based image retrieval systems (CBIR) are constantly evolving with advances in AI-based image analysis and different variants are evaluated in centrally organized competitions such as ImageCLEF^9^. The potential of CBIR systems to support radiological reporting was demonstrated for various modalities such as mammography^10^.

With the help of modern technical achievement in the field of AI-based image analysis^11^, Siemens Healthineers developed in cooperation with the investigating hospital a software for interactive image search of lung diseases.

In addition to the retrieved reference cases, the radiologist can access information about the disease in the system through an integration of the Thieme eRef database (https://eref.thieme.de/cockpits/0/0/coRadenOGW0020/0).

This study aimed to determine whether residents in radiology could provide more accurate diagnoses of chest CTs using CBIR searches without any substantial increase in the time required to make a diagnosis.

## Methods

### Ethics statement

This study was conducted in accordance with all guidelines set forth by the approving institutional ethics committee of the university hospital Essen (approval code - 20-9454-BO). Written informed consent was waived by the ethics committee of the university hospital Essen due to the retrospective character of the study. All data were completely anonymized before being included in the study.

### Study design

To examine whether SPS can increase residents' diagnostic accuracy, five residents without a specialization in chest CT scans were asked to analyze 50 lung CT scans based solely on image data. Of the five residents, two were in their first year of training, two were in their second year of training, and one was in the third year of training with 6 months, 8 months, 16 months, 18 months and 26 months of experience in reading CT scans.

For this purpose, 50 cases were presented to the residents on two occasions at baseline (t0) and at least three months later (t1) (105 days, 147 days, 123 days, 99 days, 91 days).

Reporting was done via an in-house developed web tool. In this tool, three diagnoses could be specified for each case. Upon selecting the open button, the case was opened in *syngo*.via and by clicking next, the case was saved and the next case was displayed on the screen. When the case was saved, the reading time for the respective case was stopped and saved at the same time and the timer for the next case was started while the next case was displayed.

**Figure 1:**
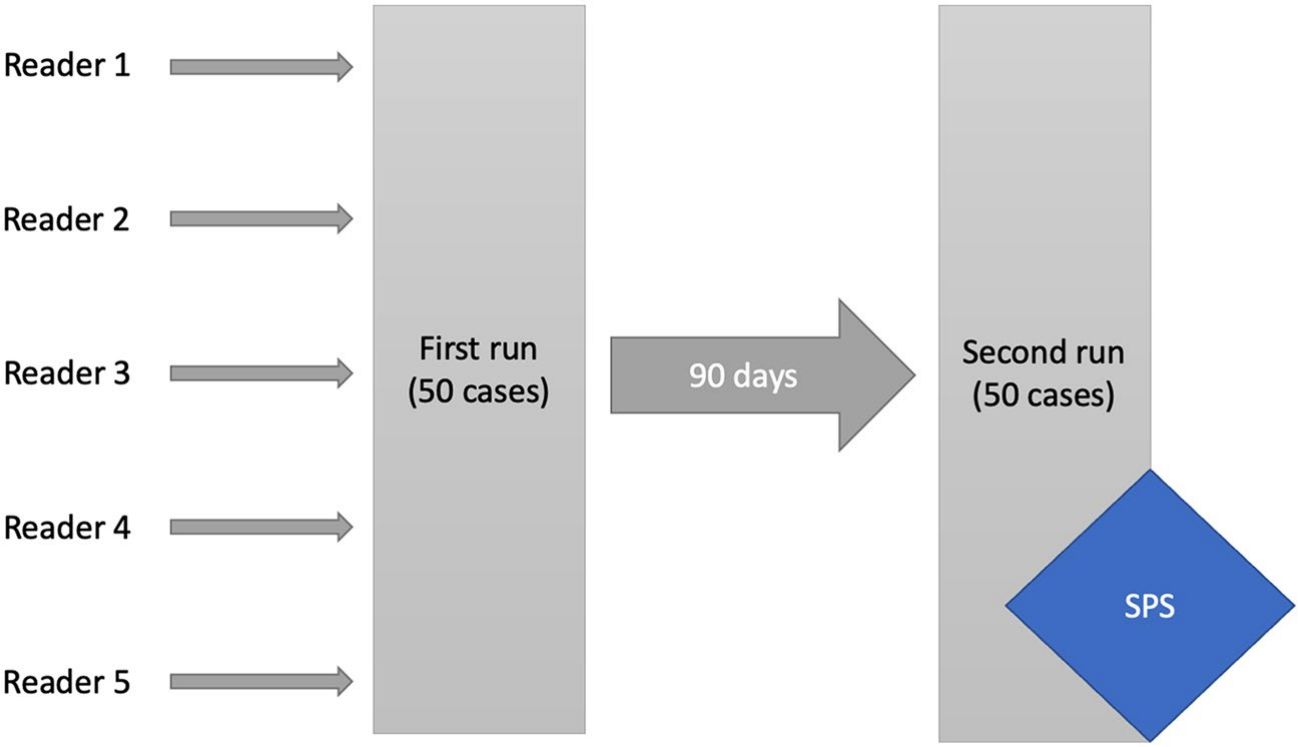
Presentation of the study design. Residents had to report the 50 cases without any tools in the first run and at least 90 days later had to report the 50 cases again with the help of SPS in the second run.

In the first run, the residents had no supporting tools, while in the second run, they were provided with SPS. After the first run, the residents were not told whether they had made correct or incorrect diagnoses. They were also asked not to talk about the cases and not to participate in training on the subject between the two phases of the study. They were also not allowed to be employed at workplaces dedicated to pneumology and thoracic surgery patients between the two evaluation rounds. In a retrospective analysis of the residents' reports between reviews, none of the diagnoses from the validation cohort were made by the residents in other patients. The study design is shown in Fig. 1.

They received a 15-min introduction to the use of SPS prior to the second run. Here, readers were demonstrated a randomly selected case from the training collective, in which they learned how the basic functions such as windowing etc. work, as well as how the SPS own functions, such as drawing the ROI, the CBIR and the knowledge database can be accessed. Utilizing this training case, they were allowed to test all functions prior to the second run.

For each case, up to three different diagnoses from a predetermined list of 85 lung diseases (shown in supplementary material 1) could be specified by the study participants. A maximum score of three points was given if only the correct diagnosis without further possible diagnoses was reported. A correct diagnosis, together with one wrong additional diagnosis scored two points, and a correct diagnosis with two wrong additional diagnoses scored one point. All cases for which no correct diagnosis was provided were granted zero points. This scoring scheme relates to the clinical routine, where each additional possible diagnosis makes the report less clear and reduces its usefulness in determining the correct diagnosis.

In an additional supplementary experiment, three raters had to reevaluate the 50 cases after a wash-out period of one year without SPS (t2) to exclude a possible bias due to the order of the evaluation. Furthermore, the residents had to reevaluate the 50 cases a fourth time (t3) with SPS to exclude a possible bias due to a gain of knowledge between the evaluations and due to the length of the wash-out period.

The differences between t1 and t2 with reversed evaluation order (first with SPS, then without SPS) are shown in Supplementary Table 1 and the differences between t2 und t3 in Supplementary Table 2. In total, therefore, there are 3 comparison scenarios:

- Without SPS (t0) -> 3-month wash-out -> with SPS (t1).
- With SPS (t1) -> 1-year wash-out -> without SPS (t2).
- Without SPS (t2) -> no wash-out -> with SPS (t3).

### Initial patient characteristics of the validation cohort

The validation cohort consisted of CTs from 50 patients examined between 2009 and 2019. Patients were, on average, 54 ± 17 years old and consisted of 42% women and 58% men. The diagnoses of the validation collective are described in Table 1.

**Table 1:** Diagnoses of the validation cohort.

N	Consensus diagnose	Accepted differential diagnosis
1	Post-primary pulmonary tuberculosis	
2	Normal	
3	Idiopathic pulmonary fibrosis	
4	(Scleroderma-related) non-specific interstitial pneumonia	
5	Lymphangioleiomyomatosis	
6	Aspergilloma	
7	Idiopathic pulmonary fibrosis	
8	Non-specific interstitial pneumonia	
9	Idiopathic pulmonary fibrosis	
10	Chronic hypersensitivity pneumonitis	
11	Aspergilloma	
12	Respiratory bronchiolitis interstitial lung disease	
13	Chronic hypersensitivity pneumonitis	
14	Chronic hypersensitivity pneumonitis	
15	Pulmonary alveolar proteinosis	
16	Pulmonary sarcoidosis—stage II	
17	Mounier-Kuhn syndrome	
18	Primary ciliary dyskinesia	
19	Lymphangioleiomyomatosis	
20	Pulmonary Langerhans cell histiocytosis	
21	Pulmonary sarcoidosis—stage II	
22	Cryptogenic organizing pneumonia	
23	Lymphocytic interstitial pneumonitis	Non-specific interstitial pneumonia
24	Post-primary pulmonary tuberculosis	
25	Allergic bronchopulmonary aspergillosis	
26	Desquamative interstitial pneumonia	
27	Mounier-Kuhn syndrome	
28	Desquamative interstitial pneumonia	
29	Desquamative interstitial pneumonia	
30	Pulmonary langerhans cell histiocytosis	
31	Pulmonary granulomatosis with polyangiitis	
32	Swyer-James syndrome	
33	Idiopathic pulmonary fibrosis	
34	Allergic bronchopulmonary aspergillosis	
35	Rheumatoid pulmonary nodule	
36	Rheumatoid pulmonary nodule	
37	Subacute hypersensitivity pneumonitis	
38	Cystic fibrosis	
39	Silicosis	
40	Silicosis	
41	Pulmonary alveolar proteinosis	
42	Pulmonary alveolar proteinosis	
43	Pulmonary sarcoidosis—stage IV	
44	Allergic bronchopulmonary aspergillosis	
45	Cryptogenic organizing pneumonia	
46	Cryptogenic organizing pneumonia	
47	Acute hypersensitivity pneumonitis	
48	Silicosis	
49	Pulmonary alveolar microlithiasis	
50	Eosinophilic granulomatosis with polyangiitis	

### Data protection

The SPS tool is cloud-based. To protect the privacy of the patients, only anonymized data was sent to and processed by the servers in the cloud. This anonymized data includes the pixel data of the slice on which the ROIs were drawn, along with further non-personal technical information (Slice Thickness, Pixel Spacing, Kernel, Manufacturer) and the outline of the ROI. All data communications with the cloud servers are routed through HTTPS and are Transport Layer Security (TLS)-encrypted. TLS is a cryptographic protocol designed to ensure the security of communications over a computer network. It is the successor to the now obsolete Secure Sockets Layer (SSL).

### Computed tomography

All CTs were performed on a Somatom Definition Flash (Siemens Healthineers), Somatom Force (Siemens Healthineers), or Somatom Definition AS + (Siemens Healthineers). All CT images were reconstructed in a hard reconstruction kernel with a layer distance and thickness of 5 mm and 1 mm, as well as a soft tissue kernel with a layer distance and thickness of 5 mm. CTs were performed at the investigating hospital between 2009 and 2019. On average, the CT dose index-volume (CTDIvol) was 8.6 ± 4.7, and the dose length product (DLP) was 283.3 ± 150.0. Patients were examined at a median of 120 kV (min. 80 kV - max. 130 kV) and an average of 119.4 + 54.2 mAs.

### Software/hardware

*Syngo*.via VB40—Advanced Visualization Software (Siemens Healthineers) was used for image interpretation. In the second study phase, the cloud-based tool Similar patient search (SPS) Web Service (Siemens Healthineers) was activated in *syngo*.via. The used Laptop was: HP(R) Mobile Workstation with Intel(R) Core i7-3920XM CPU @2.90Ghz, 32 GB RAM and 1 TB HDD with Windows Server 2019 installed as operating system. The *syngo*.via version installed on the system was modified, such that it accessed the Similar Patient Search prototype version, as the Similar patient search was not generally available at the point of time when the research was conducted.

### syngo—Similar patient search

**Figure 2:**
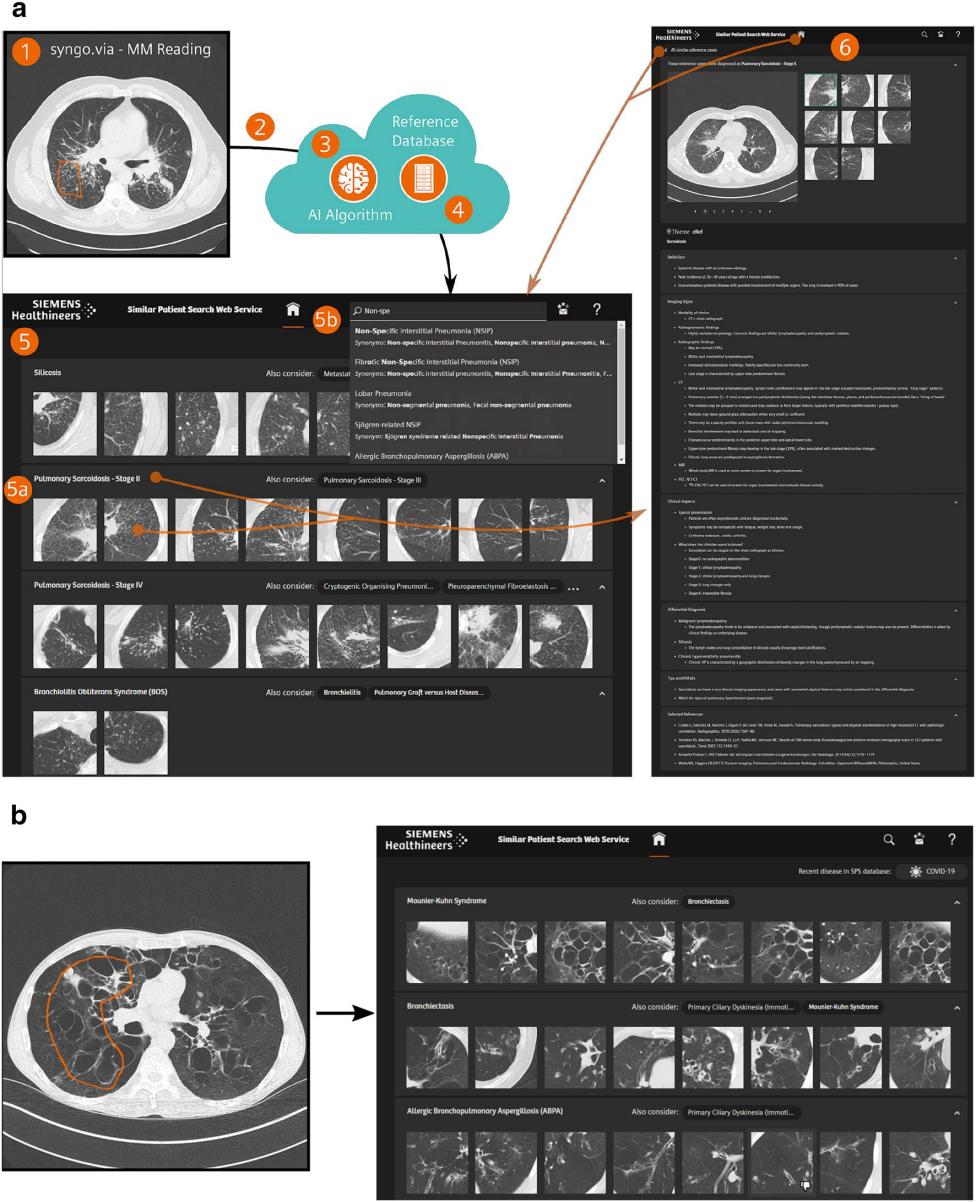
(**a**) Schematic Illustration of the Similar Patient Search Web Service. (**1**) The user selects an ROI on an axial CT slice using the host Application (*syngo*.via VA40A). The slice, along with the ROI, is sent to the cloud service (**2**). A deep-learning-based feature representation (fingerprint) for the slice/ROI combination is computed in the cloud (**3**). The fingerprint is then compared to pre-generated fingerprints from the reference database (**4**). The tool returns a webpage that shows the most similar image patches from the reference database and their corresponding diagnosis (**5**). The clinical reference content from Thieme is accessible by a single click on the disease detail page (**6**). (**b**) Example SPS result from the study: Left: Input Slice (case with Mounier Kuhn Syndrome) with the ROI chosen by the study participant (orange outline) Right: SPS User interface with top 3 results of the similarity search for this case.

The SPS is an interactive CBIR tool designed to support radiologists in their routine reading of chest CTs, with a focus on CT patterns commonly seen in ILDs. Powered by an image-based search engine, the tool enables fast access to both similar images from a large database of diagnosed cases and the related clinical reference content. A schematic illustration of the SPS is shown in Fig. 2a and an example case in Fig. 2b.

The supporting reference database of the SPS was constructed using chest CT scans from 621 patients diagnosed with one of 69 diseases that affect the lung. For each scan, axial ROIs that are most representative of the underlying disease were annotated by an expert chest radiologist. A total number of 13,658 annotated ROIs were included in the database.

The technical backbone of the SPS search engine is an image embedding function that maps a ROI to a fixed-length feature representation. The embedding function takes the form of a deep residual convolutional neural network^11^. The network was trained using metric learning techniques so that different pathological patterns are well-separated in the feature space. Given a query ROI, a feature representation is first computed using the embedding network, after which we calculate and sort the Euclidean distance between the query feature and each reference feature. Reference ROIs whose feature representations are closest to the query features are returned as top matches.

SPS is currently available for the Advanced Visualization Software *syngo*.via VB40B and higher and Syngo Carbon as the “CT Lung Assistant Tool”. A prototype was used for this study which employed the same similarity search algorithm as the one used in the product version VA12A of SPS. A video clip describing the later product version of SPS can be viewed at the following link:

https://pep.siemens-info.com/en-us/syngo-via-vb50-similar-patient-search-web-service-e-clip

The workflow of the SPS tool is the following (see Fig. 2 A for illustration):

1. The radiologist draws a 2D-ROI on an axial CT slice.
2. The CT slice and ROI are sent to the cloud server.
3. On the server, a feature representation of the CT Slice/ROI combination is generated using a neural network that was trained to differentiate between radiographic patterns in lung CTs.
4. The feature representation of the generated input data is compared to pre-generated features of the reference database, which contains 13,658 annotations on 621 cases from 69 different diseases.
5. The result of the similarity search, a list of the eight most similar diseases displayed in the *Result Overview*, is sent back to the user and presented on an interactive website
  1. *Disease result*: For each of the listed diseases, similar patches from the database are presented. A Disease Detail Page with similar patches and Thieme clinical reference content is directly accessible with a single click on the disease name, the patches, or the diseases listed in the *Also Consider* section. The *Also Consider* section provides a list of diseases that may present similar radiological patterns to the disease in question.
  2. *Textual search*: To access diseases (similar patches and Thieme clinical reference content) that are not provided in the result list but are in the SPS scope, a textual search is available.
6. *Disease detail page*: The Disease Detail Page contains similar patches from the corresponding disease and the Thieme eRef content. The clinical reference content is structured according to the following categories:
  1. Definition
  2. Imaging signs
  3. Clinical aspects
  4. Differential diagnosis
  5. Tips and pitfalls
  6. Selected references

By clicking the “Back” button or the “Home” button, the user can navigate back to the Result Overview.

Further information on SPS can be found in supplementary material 1.

### Evaluation cohort

The sample size was calculated for the binary question of whether the correct diagnosis was made. For this purpose, the accuracy of the residents without and with SPS was estimated. From our clinical experience, we estimated that for these rare conditions, the correct diagnosis is made only 20% of the time and with SPS about 50% of the time. With a confidence interval of 95% and a power of 80%, this resulted in a sample size of 36 using a sample size calculation for comparing two proportions (α = 0.05; ß = 0.2; delta = 0.3) (Wang, H. and Chow, S.-C. 2007. Sample Size Calculation for Comparing Proportions. Wiley Encyclopedia of Clinical Trials). However, because this is associated with high uncertainty due to the estimation, 50 cases were included. In the following, an experienced thoracic radiologist assembled a collective of 100 cases that covered the spectrum of complex lung diseases as broadly as possible with a confirmed diagnosis recognizable by imaging. The radiologist selected the cases from the database by means of a full-text search. No data was used from the training data set, and only patients whose diagnosis was histologically confirmed, microbiologically confirmed, or deemed confirmed at an interdisciplinary conference, were included. After the exclusion of all patients that were already included in the training collective, we randomly selected 50 cases for the evaluation cohort. Table 1 shows the diseases of the patients in the validation collective. Two radiology consultants with four and eight years of clinical experience respectively cooperatively analyzed the diagnosis of ILDs. All available clinical and imaging data were used to determine whether the diagnosis could be made unambiguously with the imaging data. One case showed no pulmonary signs of the disease, which was rated as healthy. In this case, three points were awarded if no diagnosis was given. If the case was mistakenly considered pathological with at least one diagnosis, zero points were given. In another case, the correct diagnosis was not clearly distinguishable from a different diagnosis, so here, two diagnoses were rated as correct.

### Statistical analysis

**Figure 3:**
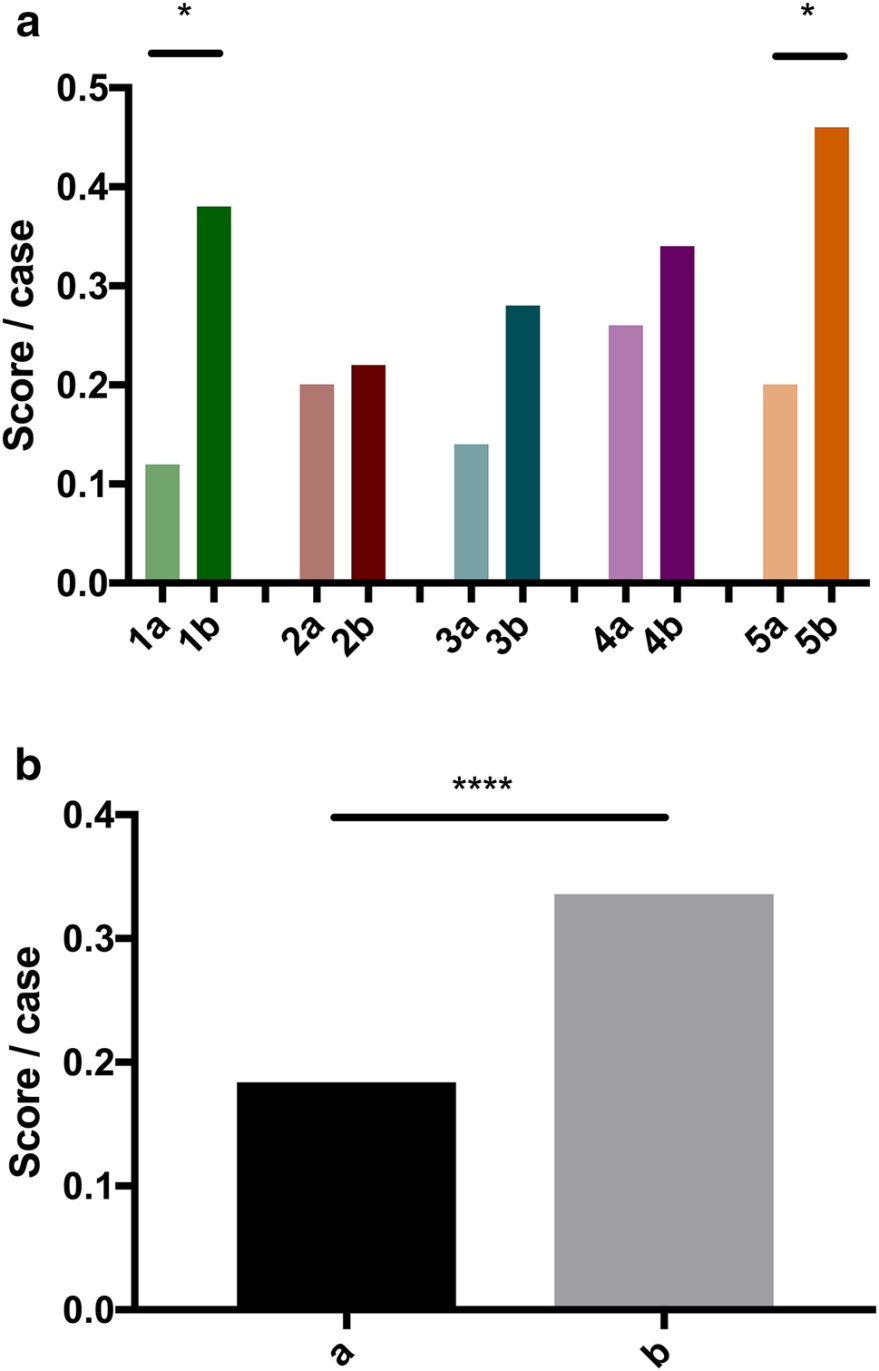
(**a**) Mean score per case of each resident 1–5 without SPS (a) and with SPS (b). (**b**) Mean score/case without SPS (a) and with SPS (b).

**Figure 4:**
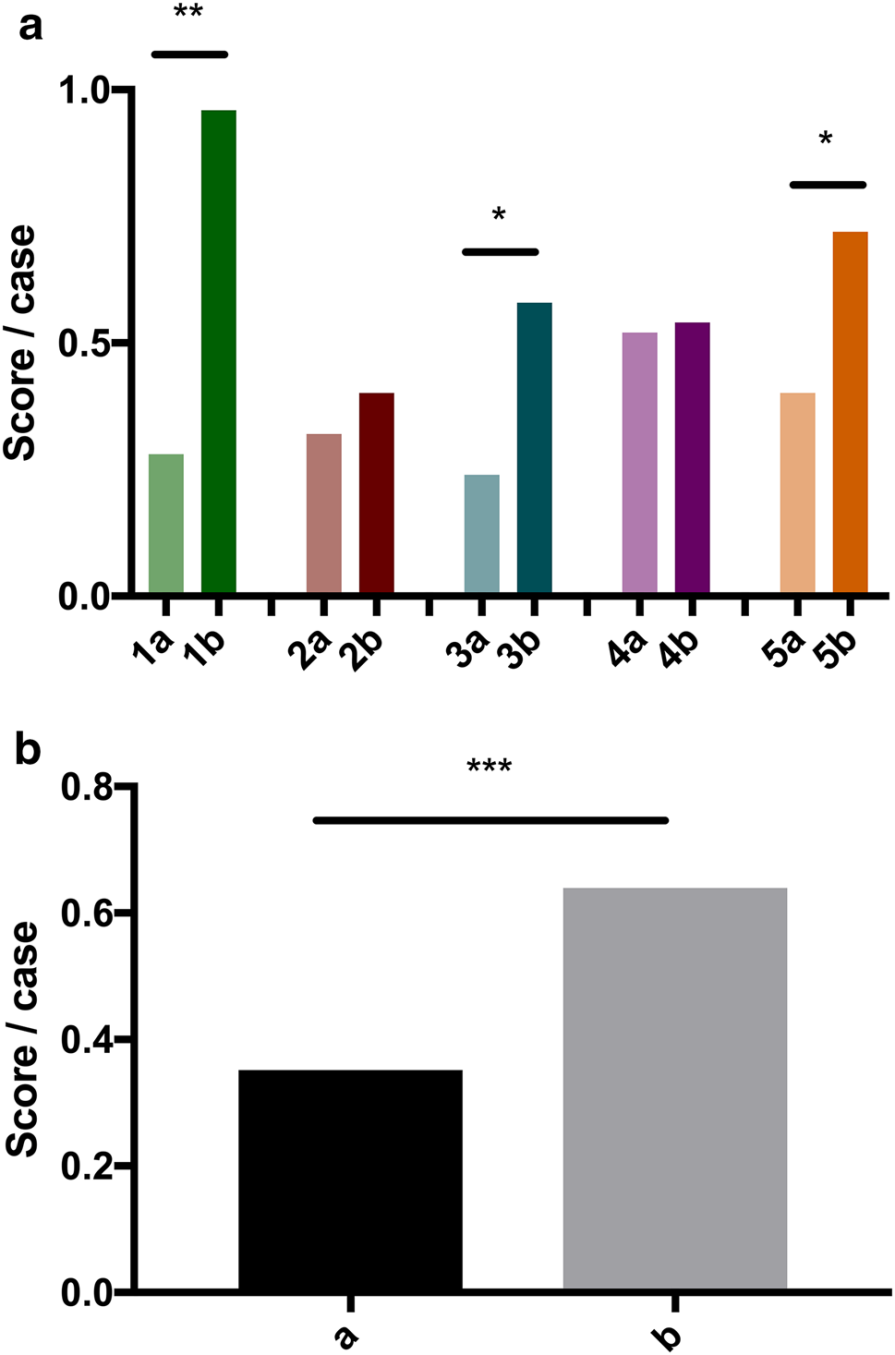
(**a**) Weighted mean score per case of each resident 1–5 without SPS (a) and with SPS (b). (**b**) Mean weighted score/case without SPS (a) and with SPS (b).

**Figure 5:**
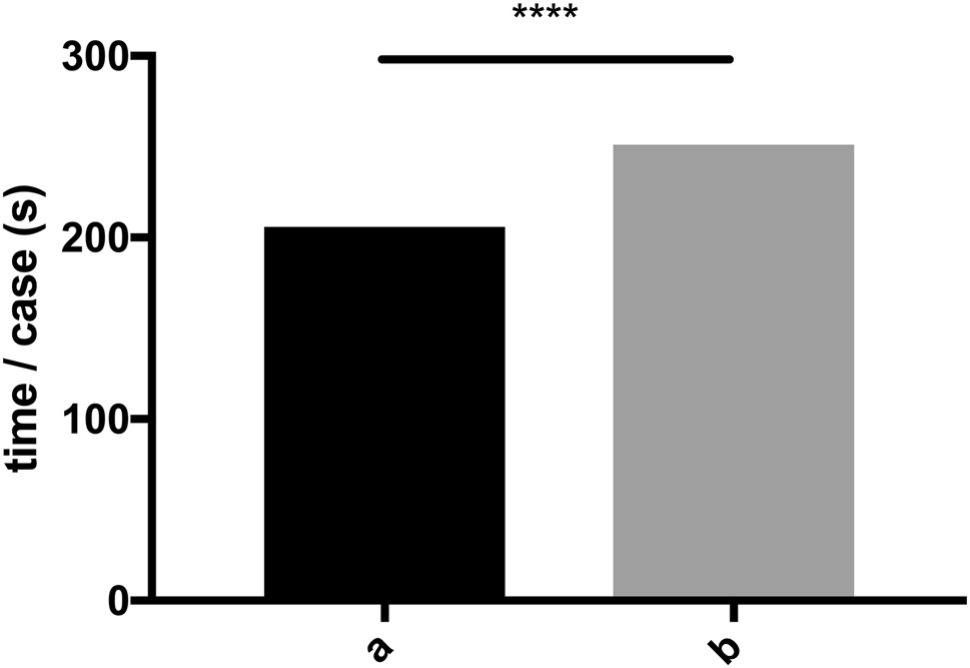
Mean time/case in seconds without SPS (a) and with SPS (b).

The statistical analyses were performed with Graphpad Prism 7 (GraphPad Software, San Diego, California, U.S.). A P-value of less than 0.05 was considered statistically significant. The cohorts were checked for normality using the Shapiro–Wilk test. The unweighted and weighted total scores of each rater were normally distributed and the significance was tested using a Student’s *t* test. The other cohorts were not normally distributed. Therefore, the Wilcoxon Matched-Pairs Signed Rank Test was used to check the scores for significant differences of paired groups (Figs. 3, 4) and the Mann–Whitney test was used for unpaired groups (Fig. 5). Fleiss kappa was used to calculate the inter-rater agreement. The intra-rater agreement was calculated using Cohen's kappa.

## Results

In the first evaluation, the residents reported the correct diagnosis in one of up to three differential diagnoses in 9.2 ± 2.5 of 50 cases (6, 10, 7, 13, 10). During the second evaluation, they were able to significantly increase their results by 82.6% (p = 0.0134) to 16.8 ± 4.1 correct diagnoses in 50 cases (19, 11, 14, 17, 23). No resident experienced a deterioration in score due to the use of SPS. Comparing the score per case of all residents in radiology, the improvement was highly significant (0.18 vs. 0.34 p < 0.0001) (Fig. 3a, b). The inter-rater agreement (Fleiss kappa) increased from 0.17 without SPS to 0.25 using SPS.

In the next step, the individual scores were weighted per case depending on whether the correct diagnosis was given without further possible diagnoses or with one or two other possible diagnoses.

In the evaluation without SPS, the residents achieved a score of 17.6 ± 5.0 out of 150 possible points (14, 16, 12, 26, 20). Using SPS, the residents were able to significantly increase their score by 81.8% (p = 0.0275) to 32.0 ± 9.5 of 150 possible points (48, 20, 29, 27, 36). No resident experienced a deterioration in score due to the use of SPS.

The improvement was highly significant when the score per case of all radiology residents is considered (0.35 vs. 0.64 p = 0.0001) (Fig. 4a, b).

All in all, the use of SPS showed a significant improvement in the diagnostic accuracy of the radiology residents. Since SPS is structured similarly to a book, which brings up the right page based on the picture, the question arises of how much time the additional research with SPS costs. Therefore, the time needed for the reading of each case was automatically recorded. The residents required an average of 205 ± 350 s per case (average total reading time of 10,250 s) without using SPS and 251 ± 220 s per case (average total reading time of 12,550 s) with SPS (Fig. 5). This corresponds to a 45 s increase, or 21.9% increase, in the time needed for the diagnosis when SPS was used. When comparing the average duration per case, the time per case was significantly higher using SPS (p < 0.0001).

**Figure 6:**
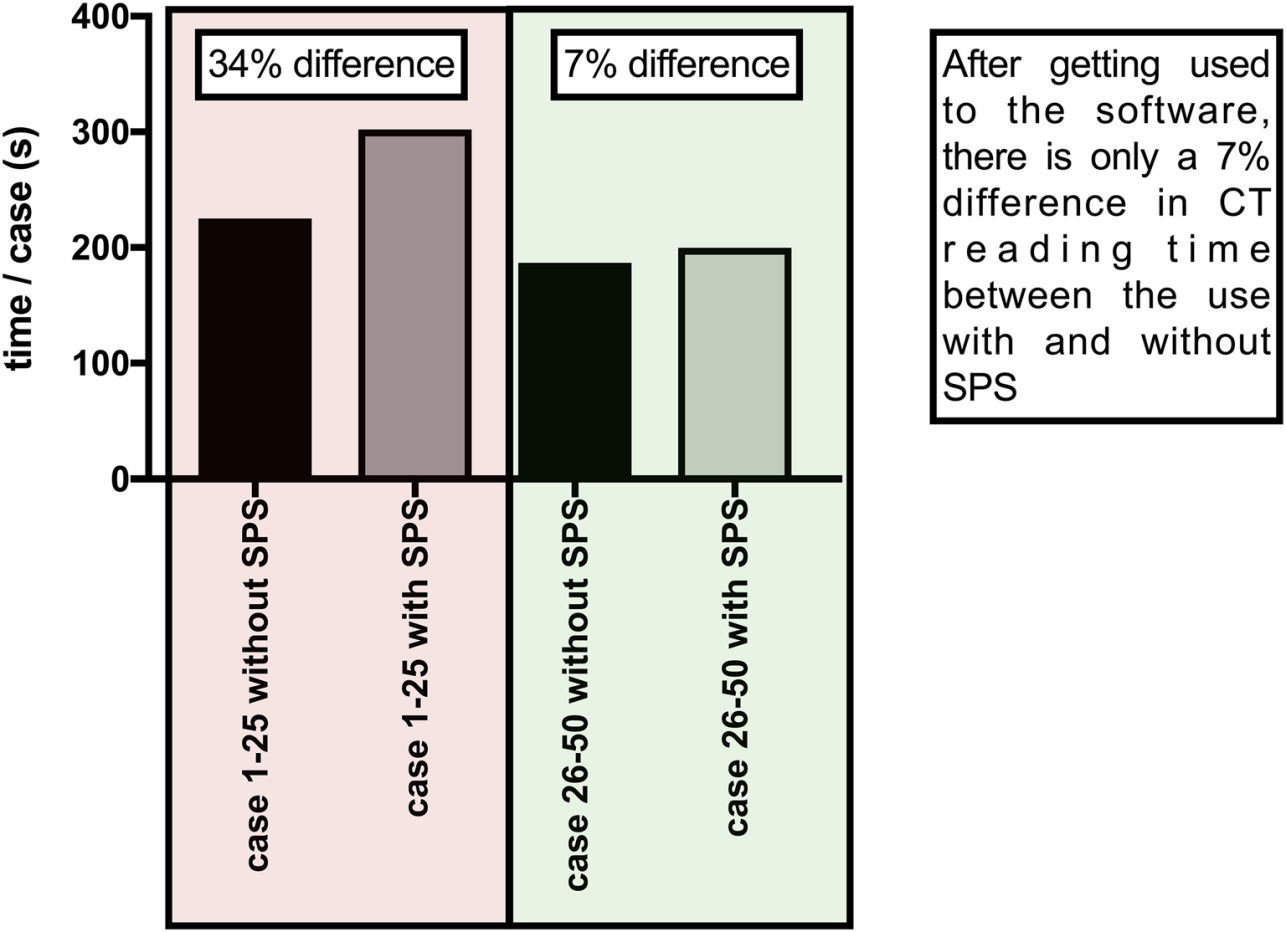
Mean time/case in seconds of the first 50% and the last 50% of cases without SPS and with SPS.

It is important to note that the residents were using SPS for the first time in the study. Whenever unknown software is used, a certain training period is required to develop familiarity with the software. In order to determine the extent to which the time increase is due to unfamiliarity with the software, we compared the time per case of the first 50% of the cases with the last 50% of the cases (Fig. 6). Both the evaluation round with SPS and without SPS showed a significant (p < 0.0001) lower time per case in the last 50% of cases compared to the first 50%. Without SPS, the time per case was reduced by 17.0% to an average of 186 s. With SPS, the time per case was reduced by 33.8% to an average of 200 s. Due to the substantial reduction in time per case in the second half of cases, the increase in time in the last 50% of cases when SPS was used was found to be only 13 s or 7.0%. The results of the respective readers are described in detail in Table 2.

**Table 2:** List of the results of the evaluation of the 5 readers (CC = R1 Correct cases (round 1); CC R2 = Correct cases (round 2); WDD R1 = Wrong differential diagnoses (round 1); WDD R2 = Wrong differential diagnoses (round 2); EDD R1 = Empty differential diagnoses (round 1); EDD R2 = Empty differential diagnoses (round 2); MC R1 = Missed cases (round 1); MC R2 = Missed cases (round 1); WSC R1 = Weighted score (round 1); WSC R2 = Weighted score (round 2); TPC R1 = Time per case (round 1); TPC R2 = Time per case (round 2); ACC R1 = Accuracy (round 1); ACC R2 = Accuracy (round 2); k = Intra-rater agreement (Cohen’s kappa).

	CC R1	CC R2	WDD R1	WDD R2	EDD R1	EDD R2	MC R1	MC R2	WSC R1	WSC R2	TPC R1	TPC R2	ACC R1	ACC R2	k
Rater 1	5	19	67	50	78	81	45	31	14	48	183.24	239.68	0.1	0.38	0.07
Rater 2	10	11	92	93	48	46	40	39	16	20	130.5	324.72	0.2	0.22	0.22
Rater 3	7	14	95	85	48	51	43	36	12	29	135.34	136.22	0.14	0.28	0.12
Rater 4	12	17	83	99	55	34	38	33	26	27	256.78	258.76	0.24	0.34	0.34
Rater 5	9	23	83	91	58	36	41	27	20	36	323.74	295.7	0.18	0.46	0.45
Mean	8.60	16.80	84.00	83.60	57.40	49.60	41.40	33.20	17.60	32.00	205.92	251.02	0.172	0.336	
SD	2.70	4.60	10.91	19.44	12.32	18.90	2.70	4.60	5.55	10.61	83.15	72.10	0.054	0.092	

## Discussion

Our study evaluated the influence of SPS on the accuracy of radiology residents in reading rare lung diseases in computed tomography. With the use of SPS, the residents’ scores increased by 82.6% to 16.8 points. Furthermore, the score weighted by the amount of differential diagnoses improved by 81.8% to an average of 32 points. The inter-rater agreement (Fleiss kappa) increased from 0.17 to 0.25. Overall, the use of SPS significantly improved the diagnostic accuracy of the radiology residents from 0.17 to 0.34.

The diagnosis of complex lung diseases requires a radiologist with a great amount of experience^12^. Furthermore, since even experienced radiologists struggle to find a reliable diagnosis in some ILDs, interdisciplinary boards are considered the gold standard for diagnosis^13^. Without any technological support, the residents were only able to make a correct diagnosis in one of 3 possible diagnoses in 9.2 of 50 cases (18.4%). If the results are balanced, so that additional but incorrect diagnoses lead to fewer points, the residents scored only 17.6 out of 150 possible points (11.7%).

This fits well with the literature, which describes that the most common type of pulmonary fibrosis is delayed on average by one year due to a frequent failure to recognize the underlying pattern^1^. Therefore, methods are urgently needed to improve the diagnostic accuracy of rare and complex lung diseases.

Current approaches have shown that convolutional neural networks can be used to detect underlying patterns of ILDs, such as ground-glass opacities or honeycombing^2,4,14^. However, these are only a small number of possible patterns that do not correspond to any singular diagnosis.

An alternative approach to improve diagnostic accuracy is to use teleradiology to ask experienced radiologists for a second opinion^15^. However, radiologists with expertise in ILDs are a limited resource, both with respect to their numbers and their availability.

Nonetheless, even with SPS, the residents were unable to identify the correct diagnosis in many cases. This may have been due to missing clinical information. To ensure the diagnosis is based purely on the image information, no clinical information other than age and gender was provided. These are essential in everyday clinical practice to make a reliable clinical diagnosis. However, these clinical data were excluded as possible confounders in this study since the quality of the clinical data also influences the quality of the report^16^. As a consequence, the total score may not represent clinical reality, but it shows the unbiased effect of SPS in terms of improving the ability of residents to reach the correct diagnosis.

Moreover, though the ability to analyze a target structure is an advantage of SPS, it is also a limitation. This is because the evaluation itself similarly depends on delineating a target structure of particular importance to the diagnosis.

However, SPS is not intended to process entire CT examinations on its own, thereby automating the diagnosis process. The radiologist remains in the driver’s seat, whilst SPS assists by presenting the radiologist with a selection of similar images and the corresponding clinical reference content.

A further limitation is that only CT scans with a slice thickness of 5 mm could be used as input to SPS at the time of evaluation. HR images, which are recommended in the guidelines for the detection of pulmonary fibrosis^17^, could not be used as an input. This is because to compile the reference database; older image material had to be used for some extremely rare diseases. At that time point, these images had only been archived in 5 mm slice thickness. On the one hand, this is a disadvantage and limitation, as it would be expected that SPS will provide a higher predictive power with HR data and in the case of sequential CT acquisition, it could not work with the examination. On the other hand, this allows better generalizability of SPS, as it delivers adequate results with standard CT data with a slice thickness of 5 mm, which can be reconstructed in any CT examination acquired in spiral mode. In the context of data augmentation, it would be conceivable to train the network in a future update to work with both HR data and normal CT data^18^.

Given the continuously increasing number of CT scans worldwide^19^, time is an important factor for reporting. The additional time required for a diagnosis because of SPS usage was reduced from an average of 77 s for the first 25 cases to only 13 s for the last 25 cases. The reduction in additional time required indicates a steep learning curve. This might be based on learning how to use the software on the one hand and on learning the disease pattern through the software on the other hand. It is also worth noting that in the clinical setting, residents would have likely searched for information in a book or on the internet, which would also have required a substantial amount of additional time.

Nevertheless, this is also a limitation regarding the accuracy of the residents; with other available additional sources of information, which are usually available in everyday clinical practice, the residents would probably have had a higher accuracy. Whether SPS improves resident accuracy more than some well-written books cannot be answered by this study. Therefore, further studies should investigate how SPS compares with other sources of information. However, given the heterogeneous landscape of books and websites, no confounder was introduced in this first study by using a potentially poor source of information.

As a further limitation, the diagnosis can theoretically be deflected from a correct diagnosis to an incorrect one by presenting similar-looking cases with a different diagnosis to the resident. This is a phenomenon that can theoretically also occur through a literature search or consultation with another colleague. Therefore, it was important to show that all residents improved on average while using SPS as an aiding tool. Overall, on average, the score was improved by more than 80% and no resident scored worse with SPS than without SPS, so this phenomenon had no major impact on the reporting in our study.

As another limitation, the sequence of evaluations might have introduced a sequence bias. To rule out the possibility that the improvements attributed to SPS were caused by a sequence bias or possible learning effects between the evaluation rounds, we performed another evaluation round without SPS after one year. Here, no significant increase could be shown between round 2 (with SPS) and round 3 (without SPS). In addition, a possible bias associated with learning effects and a potential bias due to the length of the wash-out period was further excluded by requiring the residents to evaluate the cases twice on the same day (round 3, round 4). Between these time points, the use of SPS again resulted in a significant increase in residents scores. The length of the wash-out period showed no correlation with changes in scores between the evaluation rounds.

Thus, the increase in points from round 1 to round 2 cannot be explained by the sequence, length of the wash-out period or by the increase in knowledge between the evaluation rounds.

Last but not least, the impact of SPS on the variation of reporting time with and without SPS could be influenced by a certain recognition value. To evaluate the influence of SPS on reporting time, follow-up studies including competing sources of reference such as textbooks are needed.

## Conclusion

Overall, the results of our validation underline the potential of a content-based image retrieval system and an integrated knowledge database (SPS) to improve the accuracy of radiology residents in CT reading of complex lung diseases.

## Supplementary Information

Supplementary Table 1.

Supplementary Table 2.

## Data Availability

The datasets used for the study are available from the corresponding author upon reasonable request.

## Abbreviations

CBIR: Content-based image retrieval
CT: Computed tomography
CTDIvol: CT dose index-volume
DLP: Dose length product
HRCT: High-resolution computed tomography
ILDs: Interstitial lung diseases
IPF: Idiopathic pulmonary fibrosis
NSIP: Nonspecific interstitial pneumonia
ROI: Region of interest
SPS: Similar patient search
TLS: Transport layer security
UIP: Usual interstitial pneumonia

## Code availability

The study was performed using the commercially available software solution SPS (Siemens Healthineers). The code for the above mentioned in-house developed webtool for reporting can be provided upon reasonable request by the corresponding author.
