# Supplementary material for: AI co-pilot: content-based image retrieval for the reading of rare diseases in chest CT
Source: Sci Rep. 2023 Mar 16;13:4336. doi: 10.1038/s41598-023-29949-3 (PMC10020154; doi:10.1038/s41598-023-29949-3)
Supplement: Supplementary file 1 — Supplementary Table 1. [file 41598_2023_29949_MOESM1_ESM.docx]

**Supplementary Table 1. Score / case and weighted score / case of Rater 1-3 of evaluation 2 (R2) and 3 (R3)**

|  | *Score / case R2* | *Score / case R3* | *Weighted score / case R2* | *Weighted score / case R3* |
| --- | --- | --- | --- | --- |
| *Rater 1* | 0.38 | 0.34 | 0.96 | 0.60 |
| *Rater 2* | 0.46 | 0.56 | 0.72 | 1.36 |
| *Rater 3* | 0.34 | 0.18 | 0.54 | 0.46 |
| *mean* | 0.39 | 0.36 | 0.74 | 0.81 |
| *SD* | 0.49 | 0.48 | 1.1 | 1.15 |
| *P-value* | - | 0.59 | - | 0.61 |
