# Supplementary material for: AI co-pilot: content-based image retrieval for the reading of rare diseases in chest CT
Source: Sci Rep. 2023 Mar 16;13:4336. doi: 10.1038/s41598-023-29949-3 (PMC10020154; doi:10.1038/s41598-023-29949-3)
Supplement: Supplementary file 2 — Supplementary Table 2. [file 41598_2023_29949_MOESM2_ESM.docx]

**Supplementary Table 2. Score / case and weighted score / case of Rater 1-3 of evaluation 3 (R3) and 4 (R4)**

|  | *Score / case R3* | *Score / case R4* | *Weighted score / case R3* | *Weighted score / case R4* |
| --- | --- | --- | --- | --- |
| *Rater 1* | 0.34 | 0.40 | 0.60 | 0.72 |
| *Rater 2* | 0.56 | 0.64 | 1.36 | 1.38 |
| *Rater 3* | 0.18 | 0.40 | 0.46 | 1.02 |
| *mean* | 0.36 | 0.48 | 0.81 | 1.04 |
| *SD* | 0.48 | 0.50 | 1.15 | 1.17 |
| *P-value* | - | 0.0039 | - | 0.0037 |
